# *In Vivo* Measurement of Brain GABA Concentrations by Magnetic Resonance Spectroscopy in Smelters Occupationally Exposed to Manganese

**DOI:** 10.1289/ehp.1002192

**Published:** 2010-09-28

**Authors:** Ulrike Dydak, Yue-Ming Jiang, Li-Ling Long, He Zhu, Jian Chen, Wen-Mei Li, Richard A.E. Edden, Shuguang Hu, Xue Fu, Zaiyang Long, Xue-An Mo, Dieter Meier, Jaroslaw Harezlak, Michael Aschner, James B. Murdoch, Wei Zheng

**Affiliations:** 1 School of Health Sciences, Purdue University, West Lafayette, Indiana, USA; 2 Department of Radiology and Imaging Sciences, Indiana University School of Medicine, Indianapolis, Indiana, USA; 3 Department of Health Toxicology, School of Public Health, Guangxi Medical University, Nanning, China; 4 Department of Radiology, First Affiliated Hospital, Guangxi Medical University, Nanning, China; 5 Russell H. Morgan Department of Radiology and Radiological Science, The Johns Hopkins University School of Medicine, Baltimore, Maryland, USA; 6 F.M. Kirby Research Center for Functional Brain Imaging, Kennedy Krieger Institute, Baltimore, Maryland, USA; 7 Guizhou Institute of Occupational Safety and Health, Zunyi, China; 8 Philips Healthcare, Guangzhou, China; 9 Department of Neurology, First Affiliated Hospital, Guangxi Medical University, Nanning, China; 10 Institute for Biomedical Engineering, University and ETH (Swiss Federal Institute of Technology), Zurich, Switzerland; 11 Division of Biostatistics, Indiana University School of Medicine, Indianapolis, Indiana, USA; 12 Department of Pediatrics, Vanderbilt University, Nashville, Tennessee, USA; 13 Toshiba Medical Research Institute USA, Mayfield Village, Ohio, USA

**Keywords:** GABA, imaging, manganese, metabolism, MRI, MRS, NAA, occupational health, parkinsonism, smelters

## Abstract

**Background:**

Exposure to excessive levels of manganese (Mn) is known to induce psychiatric and motor disorders, including parkinsonian symptoms. Therefore, finding a reliable means for early detection of Mn neurotoxicity is desirable.

**Objectives:**

Our goal was to determine whether *in vivo* brain levels of γ-aminobutyric acid (GABA), *N*-acetylaspartate (NAA), and other brain metabolites in male smelters were altered as a consequence of Mn exposure.

**Methods:**

We used T1-weighted magnetic resonance imaging (MRI) to visualize Mn deposition in the brain. Magnetic resonance spectroscopy (MRS) was used to quantify concentrations of NAA, glutamate, and other brain metabolites in globus pallidus, putamen, thalamus, and frontal cortex from a well-established cohort of 10 male Mn-exposed smelters and 10 male age-matched control subjects. We used the MEGA-PRESS MRS sequence to determine GABA levels in a region encompassing the thalamus and adjacent parts of the basal ganglia [GABA-VOI (volume of interest)].

**Results:**

Seven of 10 exposed subjects showed clear T_1_-hyperintense signals in the globus pallidus indicating Mn accumulation. We found a significant increase (82%; *p* = 0.014) in the ratio of GABA to total creatine (GABA/tCr) in the GABA-VOI of Mn-exposed subjects, as well as a distinct decrease (9%; *p* = 0.04) of NAA/tCr in frontal cortex that strongly correlated with cumulative Mn exposure (*R* = −0.93; *p* < 0.001).

**Conclusions:**

We demonstrated elevated GABA levels in the thalamus and adjacent basal ganglia and decreased NAA levels in the frontal cortex, indicating neuronal dysfunction in a brain area not primarily targeted by Mn. Therefore, the noninvasive *in vivo* MRS measurement of GABA and NAA may prove to be a powerful tool for detecting presymptomatic effects of Mn neurotoxicity.

Manganese (Mn) is an essential trace element important for neuronal function; however, excessive exposure to Mn has been associated with a severe movement disorder known as manganism. The clinical manifestations of Mn poisoning pertain to an extrapyramidal syndrome with a pattern similar, but not identical, to Parkinson’s disease (PD). The signs and symptoms frequently include action tremor with absence or low level of resting tremor, hypertonia, and adiadochokinesia. In severe cases, gait disturbance is observed. Furthermore, Mn neurotoxicity has been linked to distorted mental function, including memory loss, apathy, and even psychosis (Aschner et al. 2007; Calne et al. 1994; Crossgrove and Zheng 2004; Jiang et al. 2006). Occupational exposure to Mn, such as from ore extraction and processing, steel and alloy production, welding, chemical synthesis, ceramic production, and dry-battery fabrication, is a common source of Mn poisoning. Because Mn-induced neurotoxicity is an important occupational health risk, there is an urgent demand for establishing effective techniques for early diagnosis of manganism. We hypothesize that pathogenic markers of Mn neurotoxicity reflecting metabolic or excitotoxic insults, such as changes in neurotransmitter levels or other brain metabolites, likely occur before structural damage.

Mn is known to accumulate most prominently in the globus pallidus (Dorman et al. 2006; Guilarte et al. 2006a; Jiang et al. 2007). It can be visualized by increased signal intensity in the globus pallidus of occupationally exposed workers in T_1_-weighted magnetic resonance imaging (MRI) (Dietz et al. 2001; Jiang et al. 2007; Kim et al. 1999; Lucchini et al. 2000; Nelson et al. 1993). A common approach to quantifying this change in signal contrast is the definition of the pallidal index (PI), the ratio between the signal intensity within the globus pallidus and a control region, commonly chosen as subcortical frontal white matter (Krieger et al. 1995). However, brain regions outside the basal ganglia, such as frontal cortex, have also been reported to be affected by Mn and to exhibit neurodegenerative changes (Guilarte et al. 2008).

To investigate the effects of Mn on brain chemistry, we used magnetic resonance spectroscopy (MRS) to measure the concentrations of the metabolites *N*-acetylaspartate (NAA; a marker of general neuronal function), total creatine (tCr; involved in energy metabolism), choline-containing compounds (Cho; an indicator of cell membrane integrity), myo-inositol (mI; a glial cell marker), and glutamate (Glu; an excitatory neurotransmitter). Furthermore, MEGA-PRESS (Mescher et al. 1998), a special MRS editing technique, allows for enhanced assessment of the small resonances of γ-aminobutyric acid (GABA) (Edden and Barker 2007).

Several *in vitro* and *in vivo* rodent studies have linked increased Mn exposure to alterations in concentrations and metabolism of neurotransmitters, in particular, dopamine, GABA, and Glu (Burton and Guilarte 2009). Findings of GABAergic alterations are conflicting. Early *in vitro* studies reported increased striatal GABA levels in Mn-exposed rodents (Bonilla 1978; Gianutsos and Murray 1982); these are supported by later work that correlates Mn exposure with increased brain GABA concentrations (Gwiazda et al. 2002; Lipe et al. 1999; Reaney et al. 2006). Other groups, however, have failed to corroborate these studies, reporting decreased GABA levels upon Mn exposure (Brouillet et al. 1993; Chandra et al. 1982; Erikson et al. 2002; Seth et al. 1981) or no association between GABA levels and Mn exposure (Bonilla et al. 1994).

In nonhuman primates with high exposure to Mn, Guilarte et al. (2006b) found an intact but dysfunctional nigrostriatal dopaminergic system. Subsequent studies in nonhuman primates found no evidence that chronic Mn exposure alters total levels of dopamine, Glu, or GABA (Burton et al. 2009; Struve et al. 2007). In contrast, in postmortem tissue studies, elevated GABA levels in PD are well established. For example, Kish et al. (1987) reported above-normal GABA levels in the PD striatum, especially in the posterior putamen, which correlated inversely with reduced dopamine levels.

The present study, to our knowledge, is the first to investigate the effect of Mn exposure on GABA concentrations in the living human brain. Our objectives were *a*) to determine the concentrations of active metabolites such as NAA (a neuronal marker) in selected brain regions of Mn-exposed subjects compared with a control group; *b*) to determine whether chronic Mn exposure in humans alters GABA levels; and *c*) to correlate all of the MRS parameters and the PI as measured by MRI to external indices of Mn exposure, such as cumulative Mn exposure.

## Materials and Methods

### Subjects, air sampling, and physiological tests

Twenty male subjects were recruited out of a large, well-studied cohort of workers from a Mn-iron (Mn-Fe) alloy factory in Zunyi, China. The average airborne MnO_2_ concentration in the current daily working environment of both the exposed and the control group, as well as the working history and medical history of all subjects, were determined in a previous study (Cowan et al. 2009). Air samples were collected over > 10 working shifts on 3 consecutive days for each work environment. Cowan et al. (2009) provided a more detailed description of the air sampling procedure and the data. Since the study by Cowan et al. (2009), the working conditions have not changed for these subjects. For the MRI and MRS study, we chose a group of 10 Mn-exposed smelters (mean age, 40.7 years; average airborne MnO_2_, 0.18 mg/m^3^; mean years of occupational exposure, 7.8 years), exposed on a daily basis at the time of the MRI study, and 10 male age-matched control subjects with no history of Mn exposure (mean age, 43.4 years; mean airborne Mn exposure, 0.003 mg/m^3^). We defined the “cumulative Mn exposure” as the product of average airborne MnO_2_ concentration of each group × years of occupational exposure × volume of inhaled air. Written informed consent was obtained from each subject before participation, and the study was approved by institutional review boards both in China and the United States.

The 20 subjects were brought to the First Affiliated Hospital of Guangxi Medical University on a weekend day, where urine and blood tests, as well as neurological and physiological tests, were performed directly before the MRI examination. In particular, Mn, Fe, and copper (Cu) levels were measured in urine, blood plasma, and red blood cells, and pulse rate and blood pressure were determined. All subjects were evaluated by a neurologist (X.A.M.), who specializes in PD, for clinical manifestation of manganism, after clinical assessment tests for PD (tremor, rigidity, bradykinesia, and postural instability) using the motor exam tests suggested by the Unified Parkinson’s Diseases Rating Scale (UPDRS) Development Team (Fahn et al. 1987). Because no symptoms were identified in any of the study participants, we did not perform UPDRS scoring in this study.

### MRI and image analysis

**Figure 1:**
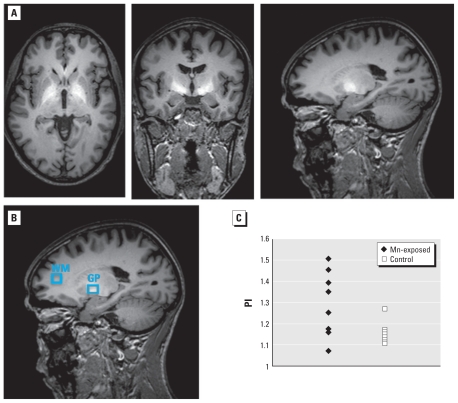
(*A*) Representative axial, coronal, and sagittal T1-weighted MRI brain images of a subject from the Mn-exposed group, depicting the hyperintense signal associated with brain Mn deposition. (*B*) ROIs used for the determination of the PI. (*C*) Distribution of the PI values for all 10 exposed subjects and all 10 control subjects. Abbreviations: GP, globus pallidus; WM, white matter.

All MRI measurements were performed on a 3 T Philips Achieva whole-body clinical scanner (Philips Healthcare, Best, the Netherlands) equipped with an eight-channel head coil. Fast T_2_-weighted images were acquired in all three orientations for exact planning of the spectroscopy volumes of interest (VOIs) and for delineation of the globus pallidus, which shows up dark in T_2_-weighted images because of its high Fe content. A high-resolution (1 mm × 1 mm × 1.5 mm^3^ pixel) T_1_-weighted three-dimensional (3D) fast- gradient echo scan [repetition time (TR) = 9.7 msec; echo time (TE) = 4.6 msec; 120 slices; field of view = 240 × 240 × 150 mm^3^; SENSE factor 2] covering the whole head was acquired for image segmentation and for detailed analysis of the areas showing hyperintensities due to Mn deposition in the brain. The resulting 3D T_1_-weighted images were reconstructed in all three dimensions and inspected for hyperintense signal in the basal ganglia (Figure 1). These images were also used to calculate the PI based on the signal ratio between a region of interest (ROI) within the globus pallidus and an ROI from white matter in the frontal lobe (Figure 1B).

### MRS, GABA editing, and spectral data analysis

**Figure 2:**
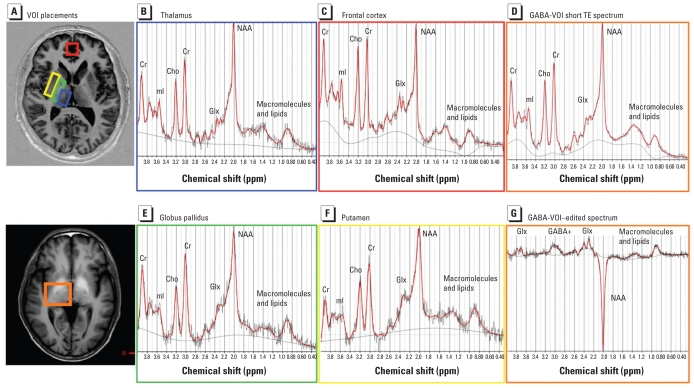
(*A*) VOI locations and (*B–F*) representative TE 30 spectra for thalamus (blue box; *B*), frontal cortex (red box; *C*), GABA-VOI [a larger basal ganglia region chosen for GABA measurements containing mainly thalamic tissue, but also putamen and globus pallidus (orange box; *D*)], globus pallidus (green box; *E*), and putamen (yellow box; *F*). (*G*) A TE 68 MEGA-PRESS difference spectrum from the GABA-VOI. All spectrum amplitudes are in institutional units, and plots were individually scaled to fill display boxes. The *x*-axis shows the chemical shift (or resonance frequency) in parts per million. The range displayed is 0.3–4.0 ppm.

In addition to imaging, short-TE single-voxel spectra (PRESS localization; TR/TE = 1,500 msec/30 msec; CHESS water suppression) were acquired from five different VOIs in each subject: frontal cortex (8 mL VOI, 96 averages), putamen (4 mL VOI, 128 averages), globus pallidus (4.5 mL VOI, 176 averages), thalamus (4.5 mL VOI, 128 averages), and a larger voxel, the GABA-VOI, centered on the thalamus but also containing the posterior parts of the putamen and globus pallidus, as well as parts of the substantia nigra (26.25 mL, 32 averages) [see Supplemental Material, Figure 1 (doi:10.1289/ehp.1002192)]. Figure 2A shows the locations of the five VOIs. For each voxel, a reference spectrum was acquired without water suppression that was later used for phase and frequency correction of the corresponding water-suppressed spectrum. Shimming and other preparation phases were performed fully automatically, resulting in line widths of < 10 Hz for the unsuppressed water peak for all spectra.

The larger volume of the GABA-VOI was necessary for the detection of GABA concentrations on the order of 1 mM. The current state-of-the-art technique to detect metabolites such as GABA, whose MRS peaks are overshadowed by much larger peaks in conventional MRS, is the homonuclear MEGA-PRESS J-editing sequence (Mescher et al. 1998). This technique, when adapted for GABA (Edden and Barker 2007; Waddell et al. 2007), eliminates most of the overlapping signal and allows for much more accurate detection of GABA. The MEGA-PRESS technique and subsequent processing are described in detail in the Supplemental Material (doi:10.1289/ehp.1002192). MEGA-PRESS optimized for GABA detection (TR = 2,000 msec; TE = 68 msec) has been validated in several clinical studies (e.g., Bhagwagar et al. 2007; Edden et al. 2009; Öz et al. 2006; Tayoshi et al. 2010) and was included in the present study in addition to the short TE single-voxel scans to investigate changes in GABA concentrations. We acquired 256 averages, half with the MEGA-PRESS editing pulse centered at 1.9 ppm and half with the pulse centered at 7.6 ppm in an interleaved fashion. See Supplemental Material, Figure 2 (doi:10.1289/ehp.1002192) for a representative GABA-edited spectrum compared with the short TE spectrum from the same VOI.

All MRS data processing was performed with LCModel (Provencher 1993), a spectral quantification tool that fits each spectrum as a weighted linear combination of basis spectra from individual brain metabolites. LCModel also provides an estimated relative standard deviation (%SD) for each metabolite as a measure of the believability of the concentration values reported. It has recently been used with MEGA-PRESS to measure GABA concentrations in schizophrenics (Tayoshi et al. 2010) and in depressed and bipolar subjects (Bhagwagar et al. 2007). For the short-TE data, we used a basis set of *in vitro* spectra from individual metabolite solutions; only fitting results with %SD < 25% were used for further statistical analysis. For the MEGA-PRESS spectra, basis sets were generated from density matrix simulations of the sequence using published values for chemical shifts and J-couplings (Govindaraju et al. 2000). The difference basis set included GABA, Glu, glutamine, glutathione, and NAA.

A known issue with estimating GABA concentrations from difference spectra is the presence of coedited macromolecule (MM) signal at 3.0 ppm, a broad signal that underlies the GABA resonance at this frequency, creating a potential source of error. We used two different approaches to model its effect and obtain GABA concentrations, yielding estimates for upper and lower limits on the “true” GABA concentration. For method 1, we did not explicitly include the potential confound of an MM signal at 3.0 ppm in the LCModel processing (the approach used by Tayoshi et al. 2010). The resulting LCModel fits have satisfyingly small %SDs for GABA, but the concentrations are probably overestimated. For method 2, an extra Gaussian peak at 3.0 ppm (“MM30”) was added to the LCModel calculation to explicitly fit the macromolecular signal (a simpler version of the technique used by Bhagwagar et al. 2007).

For all spectra (PRESS as well as MEGA-PRESS), metabolite levels were expressed as a ratio of metabolite to tCr. In the case of the GABA-edited spectra, the reference values for tCr were taken from the average of the spectra with the MEGA-PRESS pulse centered at 7.6 ppm (thus not affecting the tCr peak at 3:02 ppm).

### Statistics

All data are expressed as mean ± SD. We compared means between Mn-exposed and control groups using a two-tailed Student *t*-test with Welch correction for unequal variances. Correlation analysis was conducted and Spearman coefficients were obtained accordingly using SPSS/PC+ for Windows (version 13.0; IBM Corporation, Somers, NY, USA).

## Results

No study participant showed clinical symptoms or signs of PD or manganism in any of the tests performed. All 20 subjects were able to finish the full study, and all imaging and spectroscopy data acquired were of adequate quality to be included in the analysis. Of the various internal exposure parameters (Fe, Mn, and Cu in blood and urine), only Cu in urine showed a significant group difference between the smelters and the control group (*p* = 0.013), with the smelters having higher levels. All other measures had group difference *p*-values > 0.1; see Supplemental Material, Table 1 (doi:10.1289/ehp.1002192).

### MRI and PI

Seven of 10 exposed subjects showed a clearly hyperintense signal in the 3D T_1_-weighted images, revealing Mn deposition in the brain. As illustrated in Figure 1, the T_1_ shortening caused by Mn deposition was most prevalent in the globus pallidus and adjacent brain regions.

As expected, a significant group difference between the exposed and nonexposed subjects was identified for the PI (*p* = 0.007). However, no cutoff value could be established to classify subjects exclusively to the exposed or nonexposed group using the PI. For example, if a cutoff value of 1.2 is chosen, seven exposed subjects are correctly identified and only one control is wrongly identified as an Mn-exposed worker (Figure 1C).

### Short-TE MRS

**Figure 3:**
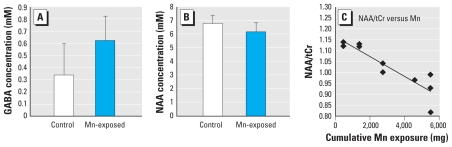
Significant metabolite concentration changes between Mn-exposed smelters and controls. (*A*) GABA concentration from the GABA-VOI quantified with LCModel using method 2, as described in “Materials and Methods” (*p* = 0.014). (*B*) NAA concentration in frontal cortex (*p* = 0.042). (*C*) Correlation between NAA/tCr in the frontal cortex and cumulative Mn exposure for the smelter group (*R* = −0.93; *p* < 0.001).

Spectral data quality was high (line width < 10 Hz, no artifacts) in all 120 short-TE spectra from the five brain locations Figure 2B–F shows typical spectra from each VOI. Table 1 lists the ratios of NAA, Cho, mI, Glu, and Glx (Glu + glutamine) to tCr as reported by LCModel from the exposed and nonexposed subjects. The only significant group difference was in the frontal cortex: NAA/tCr was significantly lower in the exposed group than in the control group (*p* = 0.04; Figure 3B), and the value decreased as the cumulative Mn exposure increased (for the Mn-exposed group alone, *R* = −0.93, *p* < 0.001; for all subjects, *R* = −0.75, *p* < 0.001; Figure 3C). Although there appeared to be a trend for a decrease in NAA/tCr in the globus pallidus of the exposed group, this decrease was not statistically significant, because 5 of 20 NAA results from these inherently broader spectra had %SD values from LCModel ≥ 25% and were thus excluded from the calculation. We found no NAA changes (i.e., group differences or correlation to other internal or external parameters of exposure indices) in thalamus or putamen, nor were the levels of other metabolites (Cho, mI, Glu, or Glx) significantly altered in any of the examined brain areas.

### GABA MRS

Figure 2G shows the LCModel fit of a GABA-edited spectrum from the 26 mL VOI centered on the thalamus (GABA-VOI). LCModel results for GABA/tCr from the MEGA-PRESS spectra are summarized in Table 1 for both method 1 (GABA fit without explicit consideration of MMs at 3.0 ppm) and method 2 (processing that included an extra macromolecular peak at 3.0 ppm). For both methods, a Student *t*-test revealed a statistically significant difference (*p* < 0.05) between the Mn-exposed and control subjects for the raw GABA signal and for GABA/tCr. The average GABA concentration was approximately 50% higher in Mn-exposed workers than in controls using method 1, and it was approximately 80% higher using method 2. Estimates for GABA concentrations from method 1, assuming [tCr] = 6.0 mM in the thalamus (Geurts et al. 2004), were 1.32 mM for the Mn-exposed group and 0.90 mM for the controls. With an MM30 model peak added in method 2, the average GABA concentration was reduced to 0.62 mM for the Mn-exposed group and to 0.34 mM for the controls (Figure 3A).

In contrast to the marked difference in GABA values between Mn-exposed and control workers, the LCModel results for the MM30 peak in method 2 were consistent for all subjects, suggesting that MM levels are not appreciably altered by Mn exposure. We saw the same consistency in the MEGA-PRESS Glu results (data not shown).

## Discussion

The nature of the involvement of GABA and Glu in Mn neurotoxicity remains unknown. This study, for the first time, employs a noninvasive MRI/MRS technique to quantify the GABA and Glu concentrations in the living brain of smelters exposed to airborne Mn in an occupational environment. Based on the data acquired, we observed that brain GABA levels are significantly increased in these workers versus matched controls in a brain volume encompassing the thalamus and parts of the basal ganglia, such as the posterior putamen and posterior parts of the globus pallidus. Furthermore, NAA levels in the frontal cortex decrease as a function of cumulative Mn exposure. Although none of the examined subjects showed any clinical symptoms of parkinsonism or motor deficits, the dramatically increased GABA levels in the thalamus and basal ganglia together with the decreased NAA levels in the frontal cortex likely reflect early metabolic or possibly pathological changes of Mn exposure.

Although the effect of Mn exposure on the GABAergic system has been investigated both *in vitro* and *in vivo* on rodent models, the results are contradictory. In nonhuman primate models, no changes in GABA were found (Burton et al. 2009; Struve et al. 2007). Notably, these studies assessed GABA levels *ex vivo*. The differences in Mn exposure paradigms, temporal relations, or species, as well as the differences in the methods used for GABA quantification, may account for the large variations in the results. In the present study, we used the MEGA-PRESS MRS technique, which allows for the specific detection of the small GABA MRS signal *in vivo*. Although MEGA-PRESS has been used for human studies by other groups (e.g., Bhagwagar et al. 2007; Edden et al. 2009, Tayoshi et al. 2010), quantification of GABA is not straightforward or well established. It is known that signal from coedited MMs contributes to the GABA peak at 3.0 ppm in the edited spectra, but because it is not trivial to eliminate this source of error completely, often only “GABA+” ( = GABA plus coedited moieties) is reported. For this study, we used a more accurate approach and fitted all of the GABA resonances in the spectrum using prior knowledge of the exact spin evolution and spectral signature of the GABA multiplets for the MEGA-PRESS sequence, and we compared two processing schemes: relying on the LCModel baseline function to handle the MM contribution at 3.0 ppm (method 1), and including a new separate MM peak in the fit (method 2). Consistently small *p*-values strongly suggest that GABA is indeed increased in the basal ganglia of Mn-exposed subjects. GABA concentrations obtained from method 2 were reduced by roughly a factor of 2 relative to method 1, in agreement with reports that the contribution of the macromolecular signal is about 50% of the total signal at 3.0 ppm (Hetherington et al. 1998; Rothman et al. 1993).

Although the mean airborne Mn level for the exposed smelters was only 0.18 mg/m^3^, which is slightly below the American Conference of Governmental Industrial Hygienists (ACGIH) threshold limit value of 0.2 mg/m^3^, we observed nearly doubled GABA concentrations in the Mn-exposed smelters in a region encompassing mainly the thalamus, but also the posterior parts of the putamen and the globus pallidus. The mechanisms associated with changes in GABA levels that are inherent to chronic Mn exposure are presently unknown. The observed increases in GABA levels corroborate observations by others of high GABA concentrations in the striatum of PD patients *ex vivo* (Hornykiewicz 2001; Kish et al. 1987) and increases in striatal GABA levels in animal models of PD, such as 6-hydroxydopamine (6-OHDA) (Bruet et al. 2003; Lindefors 1993). Changes in GABAergic neurotransmission in PD patients and 6-OHDA rats suggest that PD motor sign expression may depend on increases in striatal GABAergic neurotransmission. Striatal GABA originates from two distinct populations of neurons: medium spiny neurons expressing local and close axonal collaterals, and interneurons that strongly inhibit striatal neuron projections (Plenz 2003). Accordingly, these interneurons seem to generate a strong inhibitory feedback, decreasing striatal output activity. Although the role of GABA in manganism has yet to be clearly delineated, the observation of a Mn-induced increase in thalamic GABA levels among Mn-exposed smelters should prompt additional in-depth investigation on the mechanisms associated with this change. As recently suggested by Guilarte (2010), Mn-induced motor dysfunction reflects altered mechanisms of presynaptic dopamine release rather than its synthesis and may be distinct from mechanisms associated with PD. Given that we did not study dopamine homeostasis in the present cohort of smelters, future studies should systematically address the effects of Mn on the dopaminergic system as well, to further delineate temporal changes, or lack thereof, between GABA and other neurotransmitter systems. In addition, improving the sensitivity of GABA-edited MRS to enable the use of smaller volumes is desirable to more precisely pinpoint brain locations with altered GABA levels. The finding of no significant changes for any other metabolites in the basal ganglia is consistent with an earlier MRS study performed on Mn-exposed welders by Kim et al. (2007).

Although the frontal cortex has not been characterized as a target of Mn neurotoxicity, it has been shown that Mn also readily accumulates in this region, although to a lesser degree than in the globus pallidus (Bock et al. 2008; Dorman et al. 2006; Guilarte et al. 2008). We found a reduced concentration of NAA (a marker of neuronal viability) in the frontal cortex of smelters and an inverse correlation with cumulative Mn exposure. These results in humans corroborate the findings of neurodegenerative changes in this brain region in Mn-exposed nonhuman primates (Guilarte et al. 2008), as well as reduced NAA levels measured by MRS in the parietal cortex from the same nonhuman primates (Guilarte et al. 2006a). Furthermore, the early phase of Mn intoxication involves psychiatric and cognitive effects that might be mediated by the frontal cortex and subcortical structures (Josephs et al. 2005). Notably, NAA/tCr changes as a function of cumulative Mn exposure are observable at Mn air levels below the ACGIH limit, consistent with early presymptomatic neuronal dysfunction. These findings suggest that measurements of cortical NAA levels may prove to be a valuable biomarker of the effect of Mn exposure.

It should be noted, however, that Chang et al. (2009) studied frontal gray matter in Mn-exposed welders using MRS and, contrary to our study, observed no changes in NAA/tCr. Moreover, they found significantly reduced mI that correlated with verbal memory score and blood Mn concentrations. In contrast, we observed no other changes in metabolite ratios. Although the MRS acquisition method (short-TE PRESS sequence at 3 T), the location of the frontal cortex VOI, the apparent quality of the MRS data, and the data analysis technique (LCModel) are very similar between the two studies, other technical differences could easily account for the differences in results, especially for reported changes in metabolite concentrations of only 10–20%, as is the case for the 9% drop in NAA reported in our study and for the 20% decrease in mI reported by Chang et al. (2009).

Although the hyperintense signal in the globus pallidus is an unambiguous marker for Mn exposure in a single subject, not all exposed subjects exhibit such a hyperintense signal. Our previous study found a 78% occurrence of intensified PI in Mn-exposed workers and an 85% occurrence in highly exposed workers (Jiang et al. 2007). In the present study, we saw hyperintense signal and thus increased PI values in 7 of 10 subjects from the exposed group, which is similar to our previous findings considering the small sample size (*n* = 10). Interestingly, the three subjects not showing any hyperintense signal did not stand out by any other measure: Years of occupational Mn exposure and cumulative Mn exposure in these three subjects spanned the full range (1 year, 459 mg; 10 years, 4,588 mg; 12 years, 5,505 mg). The mechanism that either protects their brain from Mn accumulation or masks the T_1_-weighted contrast caused by Mn in the brain remains to be explored. GABA concentrations in these three subjects varied considerably, from the highest GABA level measured in all subjects to lower values (2.0 mM, 1.2 mM, and 1.6 mM, using method 1).

## Conclusions

We present for the first time *in vivo* quantification of GABA in Mn-exposed workers and show a significant, almost 2-fold increase in GABA levels compared with control subjects in a brain region containing the thalamus and parts of the basal ganglia. The fact that GABA levels seem to be largely independent of the MRI signal hyperintensity associated with Mn deposition in MRI images, and the finding that NAA decreases with exposure in the frontal cortex, where less Mn accumulates than in basal ganglia structures, both support the notion suggested by Burton and Guilarte (2009) that it is the intrinsic vulnerability to injury by Mn rather than the amount of accumulated Mn in the brain that defines its neurotoxicity. Because elevated GABA levels mirror findings in PD, and decreased NAA levels corroborate neurodegenerative changes (or at least neuronal dysfunction) observed in nonhuman primates, these findings may lead to a novel approach in identifying early presymptomatic pathogenic effects of Mn exposure. Larger sample sizes and longitudinal studies will be needed to further validate these results.

## Figures and Tables

**Table 1:** Metabolite concentration ratios from five brain regions.

						GABA/tCr^a^	
Brain region	NAA/tCr^b^	Cho/tCr^b^	mI/tCr^b^	Glx/tCr^b^	Glu/tCr^b^	Method 1	Method 2
GABA-VOI							

Smelters	1.12 ± 0.08	0.30 ± 0.02	0.68 ± 0.09	0.82 ± 0.07	0.78 ± 0.05	0.22 ± 0.06	0.10 ± 0.03
Controls	1.12 ± 0.08	0.31 ± 0.02	0.76 ± 0.14	0.82 ± 0.10	0.77 ± 0.10	0.15 ± 0.07	0.06 ± 0.04
Percent change	0	−3.2	−10.5	0	1.3	47	82^c^
*p*-Value^d^						0.028	0.014

Thalamus							

Smelters	1.01 ± 0.10	0.28 ± 0.03	0.63 ± 0.13	1.35 ± 0.28	1.01 ± 0.15		
Controls	1.00 ± 0.15	0.30 ± 0.04	0.61 ± 0.10	1.19 ± 0.35	0.90 ± 0.20		
Percent change	1.0	−6.7	3.3	13.5	12.2		

Putamen							

Smelters	0.71 ± 0.11	0.27 ± 0.04	0.53 ± 0.09	1.79 ± 0.32	1.14 ± 0.17		
Controls	0.63 ± 0.14	0.26 ± 0.02	0.48 ± 0.07	1.86 ± 0.33	1.18 ± 0.12		
Percent change	12.7	3.9	10.4	−3.8	−3.4		

Globus pallidus							

Smelters	0.75 ± 0.22	0.26 ± 0.03	0.45 ± 0.17	1.35 ± 0.49	1.03 ± 0.22		
Controls	0.63 ± 0.17	0.24 ± 0.03	0.51 ± 0.10	1.36 ± 0.37	0.99 ± 0.24		
Percent change	19.1	8.3	−11.8	−0.7	4.0		

Frontal cortex							

Smelters	1.03 ± 0.11	0.32 ± 0.04	0.93 ± 0.16	1.48 ± 0.32	1.19 ± 0.18		
Controls	1.13 ± 0.10	0.31 ± 0.02	0.88 ± 0.10	1.54 ± 0.17	1.24 ± 0.17		
Percent change	−8.9	3.2	5.7	−3.9	−4.0		
*p*-Value^d^	0.042						

a From TE 68 MEGA-PRESS spectra.

b From short-TE spectra.

c All percent changes were calculated from ratio values before rounding, in this case, 0.104/0.057.

d *p*-Values were calculated using a two-tailed Student *t*-test.
